# Supplementary material for: Discovery and application of insertion-deletion (INDEL) polymorphisms for QTL mapping of early life-history traits in Atlantic salmon
Source: BMC Genomics. 2010 Mar 8;11:156. doi: 10.1186/1471-2164-11-156 (PMC2838853; doi:10.1186/1471-2164-11-156)
Supplement: Additional file 2 — Information on developed 76 locus single-run INDEL panel in Atlantic salmon. Information on fluorescence labeling, primer concentrations, PCR pooling and links to alignments, INDEL motifs and GENESCAN (Burge and Karlin 1997) predictions of genes/exons are available in html format. [file 1471-2164-11-156-S2.ZIP › Additionalfile2/snpsummary5397.html]

```
Cluster 1368 Contig 1

prev  Summary    Contig List  next
```

Size of Consensus sequence = 1312

Number of sequences = 25

Minimum redundancy = 6

Key

A gi|117423570|gb|EG755794.1|EG755794 EST\_ssal\_plnb\_6897 ssalplnb mixed\_tissue Salmo salar cDNA Salmo salar cDNA clone ssal\_plnb\_029\_338\_fwd 3', mRNA sequence  
B gi|117546593|gb|EG878038.1|EG878038 EST\_ssal\_eve\_21595 ssaleve thyroid Salmo salar cDNA Salmo salar cDNA clone ssal\_eve\_529\_138\_rev 5', mRNA sequence  
C gi|117423615|gb|EG755839.1|EG755839 EST\_ssal\_plnb\_6937 ssalplnb mixed\_tissue Salmo salar cDNA Salmo salar cDNA clone ssal\_plnb\_029\_359\_rev 5', mRNA sequence  
D gi|117430328|gb|EG762552.1|EG762552 EST\_ssal\_sjb\_618 ssalsjb mixed\_tissue Salmo salar cDNA Salmo salar cDNA clone ssal\_sjb\_005\_024\_rev 5', mRNA sequence  
E gi|84981529|gb|DW531879.1|DW531879 EST\_ssal\_plnb\_515 plnb Salmo salar cDNA clone ssal\_plnb\_005\_086\_fwd 3', mRNA sequence  
F gi|89874451|gb|DY730574.1|DY730574 EST\_ssal\_rgb2\_86313 ssalrgb2 mixed\_tissue Salmo salar cDNA Salmo salar cDNA clone ssal\_rgb2\_641\_080\_fwd 3', mRNA sequence  
G gi|89879354|gb|DY735477.1|DY735477 EST\_ssal\_rgb2\_91216 ssalrgb2 mixed\_tissue Salmo salar cDNA Salmo salar cDNA clone ssal\_rgb2\_649\_129\_fwd 3', mRNA sequence  
H gi|24392557|gb|CA062314.1|CA062314 ssalrgb529370 mixed\_tissue Salmo salar cDNA, mRNA sequence  
I gi|84981609|gb|DW531959.1|DW531959 EST\_ssal\_plnb\_595 plnb Salmo salar cDNA clone ssal\_plnb\_006\_040\_fwd 3', mRNA sequence  
J gi|117867180|gb|EG939876.1|EG939876 EST\_ssal\_evf\_23457 ssalevf mixed\_tissue Salmo salar cDNA Salmo salar cDNA clone ssal\_evf\_530\_354\_rev 5', mRNA sequence  
K gi|117513981|gb|EG845740.1|EG845740 EST\_ssal\_eve\_58458 ssaleve thyroid Salmo salar cDNA Salmo salar cDNA clone ssal\_eve\_579\_260\_rev 5', mRNA sequence  
L gi|24354241|gb|CA048071.1|CA048071 ssalsrkb001046 kidney Salmo salar cDNA, mRNA sequence  
M gi|119020912|gb|EG355409.2|EG355409 Ss\_Kidn\_22C05\_M13 Kidney SSH library Salmo salar cDNA clone Ss\_Kidn\_22C05 5', mRNA sequence  
N gi|89829210|gb|DY691994.1|DY691994 EST\_ssal\_plnb\_4904 ssalplnb mixed\_tissue Salmo salar cDNA Salmo salar cDNA clone ssal\_plnb\_025\_084\_rev 5', mRNA sequence  
O gi|29323040|gb|CB511814.1|CB511814 ssalrgb547350 mixed\_tissue Salmo salar cDNA, mRNA sequence  
P gi|117430329|gb|EG762553.1|EG762553 EST\_ssal\_sjb\_619 ssalsjb mixed\_tissue Salmo salar cDNA Salmo salar cDNA clone ssal\_sjb\_005\_024\_fwd 3', mRNA sequence  
Q gi|117513982|gb|EG845741.1|EG845741 EST\_ssal\_eve\_58459 ssaleve thyroid Salmo salar cDNA Salmo salar cDNA clone ssal\_eve\_579\_260\_fwd 3', mRNA sequence  
R gi|117568309|gb|EG889285.1|EG889285 EST\_ssal\_evf\_47257 ssalevf mixed\_tissue Salmo salar cDNA Salmo salar cDNA clone ssal\_evf\_563\_062\_fwd 3', mRNA sequence  
S gi|84981608|gb|DW531958.1|DW531958 EST\_ssal\_plnb\_594 plnb Salmo salar cDNA clone ssal\_plnb\_006\_040\_rev 5', mRNA sequence  
T gi|117548579|gb|EG880024.1|EG880024 EST\_ssal\_eve\_23382 ssaleve thyroid Salmo salar cDNA Salmo salar cDNA clone ssal\_eve\_531\_333\_fwd 3', mRNA sequence  
U gi|45319494|gb|CK889761.1|CK889761 SGP149983 Atlantic salmon Ovaries cDNA library Salmo salar cDNA clone KG6-0292 5', mRNA sequence  
V gi|89829209|gb|DY691993.1|DY691993 EST\_ssal\_plnb\_4903 ssalplnb mixed\_tissue Salmo salar cDNA Salmo salar cDNA clone ssal\_plnb\_025\_084\_fwd 3', mRNA sequence  
W gi|117867181|gb|EG939877.1|EG939877 EST\_ssal\_evf\_23458 ssalevf mixed\_tissue Salmo salar cDNA Salmo salar cDNA clone ssal\_evf\_530\_354\_fwd 3', mRNA sequence  
X gi|85039780|gb|DW567958.1|DW567958 EST\_ssal\_rgb2\_32377 rgb2 Salmo salar cDNA clone ssal\_rgb2\_552\_159\_fwd 3', mRNA sequence  
Y gi|117546594|gb|EG878039.1|EG878039 EST\_ssal\_eve\_21596 ssaleve thyroid Salmo salar cDNA Salmo salar cDNA clone ssal\_eve\_529\_138\_fwd 3', mRNA sequence

2 SNPs detected

A B C D E F G H I J K L M N O P Q R S T U V W X Y  cosegregation weighted

776 . . . . . . . . . . . . . - T T - T - - T - - T T   2/2 48.00
777 . . . . . . . . . . . . . - A A - A - - A - - A A   2/2 48.00
